# European Web-Based Platform for Recording International Health Regulations Ship Sanitation Certificates: Results and Perspectives

**DOI:** 10.3390/ijerph15091833

**Published:** 2018-08-24

**Authors:** Varvara A. Mouchtouri, Diederik Van Reusel, Nikolaos Bitsolas, Antonis Katsioulis, Raf Van den Bogaert, Björn Helewaut, Inge Steenhout, Dion Damman, Miguel Dávila Cornejo, Christos Hadjichristodoulou, the EU SHIPSAN ACT Joint Action Partnership

**Affiliations:** 1 Department of Hygiene and Epidemiology, Faculty of Medicine, University of Thessaly, Larissa 41222, Greece; mouchtourib@med.uth.gr (V.A.M.); nmpits@med.uth.gr (N.B.); akatsioul@med.uth.gr (A.K.); 2 Seaport Cell, 2018 Antwerp, Belgium; dirk.vanreusel@gezondheid.belgie.be (D.V.R.); raf.vandenbogaert@gezondheid.belgie.be (R.V.d.B.); bjorn.helewaut@gezondheid.belgie.be (B.H.); Inge.Steenhout@gezondheid.belgie.be (I.S.); 3 Federal Environmental Inspection (Biocides and Pesticides, Dangerous Products), Federal Public Services Health, Safety of the Food Chain and Environment, Victor Hortaplein 40/10, 1060 Brussel, Belgium; dion.damman@gezondheid.belgie.be; 4 Health Control Unit Deputy Directorate General of Foreign Health Directorate General for Public Health, Quality and Innovation Ministry of Health, Consumption and Social Welfare, Paseo del Prado 18-20, 28014 Madrid, Spain; mdavila@mscbs.es

**Keywords:** international health regulations, Ship Sanitation Certificates, inspection, ship, travel, maritime health, sanitation

## Abstract

The purpose of this study was to report the data analysis results from the International Health Regulations (2005) Ship Sanitation Certificates (SSCs), recorded in the European Information System (EIS). International sea trade and population movements by ships can contribute to the global spread of diseases. SSCs are issued to ensure the implementation of control measures if a public health risk exists on board. EIS designed according to the World Health Organization (WHO) “Handbook for Inspection of Ships and Issuance of SSC”. Inspection data were recorded and SSCs issued by inspectors working at European ports were analysed. From July 2011–February 2017, 107 inspectors working at 54 ports in 11 countries inspected 5579 ships. Of these, there were 29 types under 85 flags (including 19 EU Member States flags). As per IHR (2005) 10,281 Ship Sanitation Control Exception Certificates (SSCECs) and 296 Ship Sanitation Control Certificates (SSCCs) were issued, 74 extensions to existing SSCs were given, 7565 inspection findings were recorded, and 47 inspections were recorded without issuing an SSC. The most frequent inspection findings were the lack of potable water quality monitoring reports (23%). Ships aged ≥12 years (odds ratio, OR = 1.77, 95% confidence intervals, CI = 1.37–2.29) with an absence of cargo at time of inspection (OR = 3.36, 95% CI = 2.51–4.50) had a higher probability of receiving an SSCC, while ships under the EU MS flag had a lower probability of having inspection findings (OR = 0.72, 95% CI = 0.66–0.79). Risk factors to prioritise the inspections according to IHR were identified by using the EIS. A global information system, or connection of national or regional information systems and data exchange, could help to better implement SSCs using common standards and procedures.

## 1. Introduction

International sea trade and population movements by ships can play a role in the global spread of diseases. Approximately 50,000 of the world’s total fleet of 93,161 propelled seagoing merchant ships sail internationally, and 90% of world trade is carried by the international shipping industry [1,2]. The International Health Regulations (2005) (IHR (2005)) with their regulatory functions, include certificates applicable to international travel and transport. They also include requirements for international ports, ship operators, container shippers, consignees and consignors, in order to provide a global regime for the control of public health risks internationally [3]. Operators of ships sailing on international voyages must hold a Ship Sanitation Certificate (SSC), which can be either a Ship Sanitation Control Exemption Certificate (SSCEC) or a Ship Sanitation Control Certificate (SSCC). SSCs are issued by inspectors of the competent authorities after conducting a ship inspection, and are valid for six months [3].

Under the IHR (2005), the purpose of ship sanitation inspections is to determine whether a public health risk exists on board, and to ensure the implementation of the necessary control measures [3]. Examples of such risks include: vectors at all stages of growth; animal reservoirs for vectors; rodents or other species that could carry human disease; microbiological, chemical, radiological, and other risks to human health; signs of inadequate sanitary measures and information concerning cases of disease. Both evidence of public health risks and the control measures implemented on ships are noted in the SSC. SSCs can be reviewed by inspectors at subsequent ports of call, and can, therefore, be considered a communications tool for information sharing among ports, related to the health and hygiene status on board ships sailing internationally.

An SSCC is issued by the competent authority when control measures are required and have been satisfactorily completed, noting the evidence found and the control measures taken. The competent authority issues an SSCEC if they are satisfied that the ship is free of infection and contamination, including vectors and reservoirs.

The World Health Organization (WHO) publishes the list of ports that have been authorised by each WHO State Party to issue SSCC, SSCEC only, or the extension of the SSCC for a period of one month (until the arrival of the ship to port where a new certificate may be issued) [4]. This list of ports is available online [4].

The global reference document for standards to be used when inspecting and issuing SSCs is given in the WHO “Handbook for Inspection of Ships and Issuance of Ship Sanitation Certificates” [5]. The handbook includes guidelines for preparing and conducting inspections, and issuing SSC. In particular, it contains a useful coding system, whereby each code represents evidence found during an inspection and the corresponding control measures to be taken. By using this coding system, inspectors around the world reference inspection findings using the same coding system, and the risk of misunderstanding inspection findings is minimized. WHO has further developed a learning programme on ship sanitation inspection/issuance of SSC, which is built around two core activities: the eLearning course and a face-to-face course [6]. This learning programme is designed for ship inspectors in charge of ship inspection and the issuance of SSC under the IHR (2005). It is built as a menu of options, plans, and objectives of the interested authorities for improving competencies of inspectors. The learning programme’s overall aims are to contribute to the harmonization of inspection practices at authorized ports globally; to improve the quality and consistency of inspections; and to enhance competent authorities’ abilities to protect public health, by achieving greater compliance from ship operators with SSC provisions under the IHR (2005).

Under the European Union (EU) project “Ship Sanitation Training Network” (EU SHIPSAN TRAINET) (2008–2011) a European Information System (EIS) was developed for recording inspection results and issuing SSC [7,8]. This system was upgraded in the framework of the EU joint action “The impact on maritime transport of health threats due to biological, chemical, and radiological agents, including communicable diseases” (SHIPSAN ACT) (2013–2016) [9]. Its purpose was to improve the implementation framework for SSC in Europe, and activities were supported by the WHO [10]. The system was designed to address the needs identified by surveys conducted in EU Member States (MS) under the framework of the project “Assessing the Usefulness of a EU Ship Sanitation Programme and Coordinated Action for the Control of Communicable Diseases in Cruise Ships and Ferries” (EU SHIPSAN) (2006–2008) [11]. These surveys revealed diverse approaches and practices in the conduct of inspections, differences in the qualifications/knowledge/experience of inspectors, differences in health and hygiene legislative applied standards, and a lack of communication among ports [12]. In the scope of EU SHIPSAN TRAINET and SHIPSAN ACT joint action, training programmes for EU ship inspectors and crew members were designed, including information on how to use the EIS.

The EIS aims to help competent health authorities at ports and inspectors of EU countries to: (a) conduct inspections according to common standards, as described in the WHO “Handbook for inspection of ships and issuing Ship Sanitation Certificates”; (b) record inspection results in a common EU database; (c) access and review SSC issued in previous ports of call and the inspection results; and (d) provide reports and analysis of inspection results, to improve inspections and hygiene standards on board ships.

This paper reports results of data analysis from the SSC that have been recorded in and issued through the EIS from July 2011 to February 2017, by inspectors working at ports of EU countries. To our knowledge, this paper is the first published evidence arising from inter-country databases and inspection data from the IHR (2005) SSC, that have been issued according to the standards of the WHO “Handbook for Inspection of Ships and Issuing Ship Sanitation Certificates”.

## 2. Materials and Methods 

### 2.1. Situation Analysis 

A cross-sectional survey was conducted in order to collect information on current practices for issuing ship sanitation certificates within EU MS, before beginning the design of the EIS. A questionnaire was developed consisting of 20 closed and semi-closed questions, to collect information on: (i) the ports authorised to issue SSCEC/SSCC or give extension to an existing certificate; (ii) the practices for collecting the Maritime Declaration of Health; (iii) the policies and practices in place for ship sanitation inspections and SSCEC/SSCC issuance; (iv) national information systems for information exchange with port health authorities; (v) national or local computerized systems for recording ship inspection results and/or issuing SSC; and (vi) the willingness of EU MS to use the EIS for recording ship inspection results and sharing information on ships.

The questionnaire was disseminated to the IHR and Early Warning and Response System (EWRS) National Focal Points (NFP) of 30 countries in Europe. Questionnaires were completed electronically through a specially designed form in an Acrobat Reader (PDF file) format. Where electronic completion of the questionnaire was not possible, telephone interviews were conducted. The data collection process took place from 3 February 2010 to 24 March 2010.

### 2.2. Focus Groups

Expert opinions on technical, legal, and information technology issues were collected through focus groups conducted to answer specific questions. The focus group—consisting of representatives from the World Health Organization (WHO), the French Ministry of Health, the Hamburg Port Health Centre, the Amsterdam Port Health Authority and the University of Thessaly in Greece—met in March 2010, June 2010, and January 2011. The expert focus group considered the situation analysis results described in the previous paragraph (that are also presented in the results section of this paper). Also considered by the focus group were the survey results conducted by the EU SHIPSAN project that have been published elsewhere [12,13]. Existing national frameworks and the EIS in EU MS were reviewed. WHO representatives participated in the focus group and provided advice related to the IHR (2005) requirements and the WHO “Handbook for Inspection of Ships and issuance of Ship Sanitation Certificates”. The expert focus group designed the EIS functions for recording ship inspections and issuing SSC.

### 2.3. Information System Development and Pilot Testing

The development of the software was subcontracted to a third party. The pilot phase of the EIS took place from 6 April 2011 to 6 June 2011. A test platform was developed and used for pilot purposes, hosting 15 pilot inspections [14]. Inspectors from the participating EU MS (France, Germany, The Netherlands, Greece) accessed the system and submitted real data related to inspections conducted in their ports. Comments about the functionality of the EIS from the participants in the pilot phase were recorded and incorporated into the final version of the EIS.

### 2.4. Information System Functions 

The information system is a web based application, using asp.net/MS SQL Server/RDBMS technology (ASP.NET 2/Net Framework 3.5/SQL Server 2008 R2 std 64-bit). It is hosted on a dedicated web server installed at the University of Thessaly (Greece), and it functions under a secure framework (login authentication/SSL web certificate HTTPS/antivirus and firewall).

The EIS can be accessed via a restricted area of a web platform with a username and password. It works as a database registry storing information about the stakeholders involved in procedures for ship sanitation inspections under the IHR (2005). The EIS also stores information on the ship inspection procedure itself, including: port health authorities of the participant European countries authorized to issue Ship Sanitation Certificates (SSCCs/SSCECs), or extension under the IHR (2005); central level authorities (i.e., Ministry of Health; ships; shipping companies; shipping agents; other competent organizations/authorities); classification societies; and ship inspectors. The EIS provides a field where inspectors working at different ports can communicate and send messages to all other ports’ inspectors. It further incorporates the checklists for ship inspections, according to the WHO “Handbook for Inspection of Ships and Issuance of Ship Sanitation Certificates”.

The inspection findings are recorded in the EIS according to the checklist in the WHO Handbook, categorized as “recommendations” or as “requirements” [5]. The checklist consists of 13 areas with a total of 364 coded items. There are 599 control measures and corrective actions, of which 262 are recommendations and 337 are requirements. Inspectors complete this checklist online and produce the SSCC or SSCEC, depending on their professional judgment and the Evidence Report Form (ERF). Inspectors complete the ERF, indicating any areas that were not inspected, the coded items that correspond to inspection findings, a description of inspection findings, the control measures taken, and which control measures were successfully performed or pending re-inspection [5]. Moreover, the ERF can be attached to an existing valid SSC, describing the evidence found during an inspection conducted (not for the purpose of issuing a new SSC). The ERF and SSC must be linked by stamping a seal on the SSC with the text: “SEE ANNEX”. The SSC are issued through the EIS according to Annex 3 of the IHR (2005), and the ERF according to Annex 7 in the WHO Handbook. The EIS further records potable water sample results and control measures applied during inspection. Once the SSCC or SSCEC has been finalized and issued, there is no ability to change the submitted data, except for adding the potable water sample results that are pending at the time of certificate issuance. Certificates and reports are available in both electronic and printable format. Inspectors can obtain the SSC and the ERF according to the model and logos of their own country, as these are included in the EIS. The EIS can also produce output reports from data submitted. The EIS supports the ability of data extraction (xls format) for statistical purposes.

If a ship has been registered and inspected in a previous port, inspectors can then review the ship registry, any previous certificates (SSCECs or SSCCs), and inspection results. Previous inspection results appear according to a colour-coded system, under the following categories: (a) “No Registered Inspections or No Finalized Results”; (b) “SSCEC”; (c) “SSCC With Already Applied Required Control Measures”; (d) “SSCC With Not Yet Completed Control Measures”; (e) “SSCC With Affected Conveyance” and (f) “Extension to an existing SSC”.

An overview panel displays pending activities related to the specific logged-in inspector. It includes the incoming/anchored ship(s) to the port, the inspections that are still pending, and the messages that have been sent to the port. The message board displays information that has been exchanged among inspectors. There is also a help tool with online user manuals and useful links. A sitemap provides the user with the structure of the site. An advanced search engine is available in every section of the EIS. The “news section” acts as a dashboard, whereby useful and up to date news of public health interest are published, and can be accessed by users of the platform.

The EIS has been operating since July 2011. It was upgraded in June 2016 to improve functionalities, as suggested by the users (i.e., content forms, interoperability, web interface, user roles, registries, and system functionality) [7].

### 2.5. Statistical Analysis

The data collected through the survey questionnaires were entered into a specifically designed database, using EPI Info software (version 3.5.4, CDC Atlanta, GA, USA). Descriptive analysis was conducted, and the remaining analysis used the statistical package SPSS 21.0 (IBM SPSS Inc., Armonk, NY, USA).

The inspection results data extracted from the EIS were analysed in the following manner. Qualitative variables were presented as frequencies with percentages and/or 95% confidence intervals (95% CI). For univariate analysis, a chi-square test or Fisher’s exact test was applied to associate ship characteristics, and other factors with the types of certificates calculating the relative risks (RR) and the corresponding 95% confidence intervals (95% CI). In multivariate analysis, logistic regression analysis was performed to identify independent risk factors for the types of certificates, calculating the odds ratios (OR) and the corresponding 95% confidence intervals (95% CI). The chi-square test for trend was used to assess any dose-response relationship between ordinal factors and types of certificates. Factors with a *p*-value of less than 0.2 in univariate analysis were included in the multivariate analysis. A result with a *p*-value < 0.05 was considered to be statistically significant. Ships inspected were of 29 different types, which for the purpose of analysis, were grouped into 11 categories:

A: Bulk Dry, Bulk Dry/Oil, Other Bulk Dry, Other Dry Cargo, Self-Discharging Bulk Dry; B: Passenger; C: Container, Other Activities container; D: Dredging, Non Propelled, Offshore Supply, Other Activities, Other Offshore, Research, Towing/Pushing; E: Fish Catching and Other Fishing; F: General Cargo, Passenger/General Cargo, Passenger/Ro-Ro Cargo, Ro-Ro Cargo; G: Inland Waterways Dry Cargo/Passenger and Inland Waterways Tanker; H: Liquefied Gas, Oil and Other Liquids; I: Non Merchant Ships; J: Chemical; K: Refrigerated Cargo.

### 2.6. Ethical Approval

Ownership of the results of the EU SHIPSAN TRAINET project and the SHIPSAN ACT joint action, including intellectual property rights are vested in the beneficiaries of the EU SHIPSAN TRAINET project and the SHIPSAN ACT joint action. Designated representatives of these beneficiaries have given their consent to the use and the publication of data of EIS. The authors have applied the rules of ownership and publicity as described in the grant agreement (20122103) articles I.11, II.5, 3, and II.5. This study contains information about analysis results of the IHR (2005) SSC, without including the names of persons or ships. The data analysed have been registered in the EIS, which operates according to the European legislation for personal data protection. Sole responsibility lies with the author and the Consumers, Health, Agriculture, and Food Executive Agency (CHAFEA) is not responsible for any use that may be made of the information contained therein.

## 3. Results

### 3.1. Situation Analysis Results

Twenty-seven out of the 30 countries responded to the questionnaire. Of the 26 countries that have identified the competent authorities for inspecting ships and issuing SSCs, six deal exclusively with duties related to ships, whereas 22 have parallel duties. Fifteen of the respondent countries declared that they request the SSCEC/SSCC on a routine basis (i.e., upon arrival to grant free pratique). Twelve of 27 countries (44.4%) have defined national guidelines for issuing SSCECs/SSCCs that include a checklist for inspection. Seven of 27 countries (26%) do not charge any fees for the issuance of SSCECs/SSCCs. Five of 27 countries (18.5%) have a database for recording ships’ inspection results for issuing the SSCC/SSCEC. Five of 27 countries (18.5%) require the Maritime Declaration of Health before berthing from all international arrivals to confirm the health situation on board. Twenty-four of 27 of the responding countries (88.9%) declared that they would use a European database for recording ship inspection results and sharing information on ships. One country replied that this would depend on the content of the database. Two countries that declared they will not use the database do not issue SSCECs/SSCCs because they do not have sea traffic in their territories.

### 3.2. Ship Sanitation Certificates Analysis Results

The EIS has been used by 107 inspectors working at 54 ports in 11 countries. A total of 5579 ships of 29 types, belonging to 85 different flags (including 19 EU flags) have been inspected. The mean age of ships inspected was 10.89 years (standards deviation 8.15, 25% percentiles = 5, 75% percentiles = 15, minimum = 0, maximum = 82). Descriptive analysis of ship and inspection characteristics are presented in Table 1. The ship age was known for 10,481 ships and the ship type was known for 10,630 ships.

From July 2011 to February 2017, the EIS included a total of 10,698 records, of which 10,281 are SSCECs and 296 SSCCs; 74 are extensions to existing SSCECs; and 47 are records of inspections without issuance of an SSC (Table 1). Moreover, the EIS was used for sharing 15 messages among inspectors working in different ports who have access to the EIS in order to communicate information found during inspections.

A total of 7118 water samplings were performed. For 6228 samplings, no laboratory results were recorded, while 175 samplings (2.5%) were positive and 725 samplings (10.2%) were negative.

A total of 138 out of the 10,698 inspections (1.3%) were excluded from the analysis of inspection findings, due to missing information recorded in the EIS. A total of 7078 inspections (66.2%) had no inspection findings, one inspection finding was recorded in 1904 inspections (17.8%), two to five inspection findings were recorded in 1362 SSC (12.7%), six to eleven inspection findings were recorded in 186 SSC (1.7%), while twelve to twenty-eight inspection findings were recorded in 30 inspections (0.3%).

No differences were found in the mean number of inspection findings in 2012, 2013, 2014, 2015, and 2016, which were 0.77, 0.76, 0.75, 0.75, and 0.58, respectively.

A total of 7565 inspection findings were recorded. The 10 most frequent inspection findings are listed in Table 2, and the frequency of inspection findings per ship area according to the WHO Handbook are found in Table 3.

Moreover, Table 3 presents the areas with inspection findings according to the WHO Handbook [5]. The mean number of inspection findings recorded in the EIS was equal to zero for 45 inspectors; >0 and ≤1.0 for 36 inspectors; 1.1–2.9 for 12 inspectors; and 3.0–6.7 for eight inspectors. One inspector had a mean number of findings equal to 21. For five inspectors, the mean numbers were not calculated due to missing data.

The mean and median numbers for inspection findings in the SSCC issued were 5.1 (standard deviation (SD) 4.6) and 4 (25% percentile = 2, 75% percentile = 7, minimum = 0, maximum = 28) respectively. Mean and median numbers for inspection findings of the SSCEC were 0.6 (SD 1.2) and 0.0 (25% percentile = 0, 75% percentile = 1, minimum = 0, maximum = 16). Inspections with SSCC differed significantly in terms of inspection findings (no finding, 1 finding, >1 finding), compared to inspections with SSCECs (*p*-value < 0.001).

Univariate analysis was conducted associating various factors with the type of SSC (SSCC or SSECC) held by ships, and with the risk of having ≥1 inspection findings (Table 4).

Logistic regression analysis showed that factors contributing to the holding of an SSCC were: ships aged ≥12 years and the absence of cargo at the time of inspection (Table 5). Being under an EU MS ship flag was found to be a protective factor for having findings in an inspection. Certain ship types were shown to be protective or contributing factors for having inspection findings or holding an SSCC (Table 5).

## 4. Discussion

This paper presents the results from the implementation of the IHR (2005) in Europe for SSCs. It demonstrates the benefits of the EIS in prioritizing inspections on high-risk ships, by reviewing previously-recorded inspections, monitoring trends over time (such as improvements in ship’s performance), and setting training priorities for both industry and public health authorities.

The majority of certificates issued were SSCECs (97%), with SSCCs comprising only 3% of certificates issued. This could be interpreted as an absence of public health risks for the ships inspected and, therefore, an SSCEC was issued. However, it would be interesting to conduct a more in-depth investigation into the decision process followed by inspectors, specifically whether an SSCEC or an SSCC should be issued, and on what criteria the decision made was based on. In addition to this, it would be of value to conduct a survey assessing the knowledge, attitudes, and practices of inspectors regarding inspections done for the issuance of SSCs. This survey could be expanded to include officers at ports, exploring how the type of SSC is interpreted. Previous experience gained from conferences, technical meetings, and training courses revealed that an SSCC is considered to demonstrate the presence of public health risks on a ship holding that type of certificate, even if control measures have been taken. Moreover, in some instances an SSCC is considered as a “stigma” for the ship, due to the lack of knowledge about the rules for issuance of the SSCC. In some cases, port health authorities can incorrectly interpret Article 39 of the IHR (2005) and may reject the free pratique, based on the fact that the ship holds an SSCC. In these cases, unnecessary delays have been reported (Dirk Van Reusel, personal communication). Guidance, training, and raising awareness would assist in the decision–making process for the issuance of the appropriate certificate type by inspectors, and the interpretation of information in the SSCC or the SSCEC by stakeholders. This would help in the correct application of Article 39 of the IHR (2005), which clearly states “*When control measures are required and have been satisfactorily completed, the competent authority shall issue a Ship Sanitation Control Certificate, noting the evidence found and the control measures taken*”. Consequently, it should be clear to all stakeholders that holding an SSCC would not mean the presence of public health risks, since the control measures have been satisfactorily taken and recorded in the certificate.

In about one third of inspections (33%), no inspection findings were recorded in the EIS. This could be interpreted as an absence of inspection findings at the ships inspected. It would be interesting to further examine the practices of inspectors and whether verbal reporting of findings are taking place, or if all inspection findings (including the minor ones) are recorded in the Evidence Report Form [5]. Moreover, training could be appropriate for areas that are less inspected or no/fewer inspection findings are found.

The majority of inspection findings were recorded in potable water areas (34%) and the galley, pantry, and service areas (34%), followed by food stores (15%) and medical facilities (11%). Almost no inspection findings were recorded in the following areas: child-care facilities, swimming pools and spas, engine room, sewage, ballast water, and cargo holds. The first two areas can be found mainly on passenger ships, but only a small number of them (69) were inspected. The inspection area for sewage and ballast water are inspected by port state control, and only issues related to public health are covered in the inspection for issuance of an SSC. These results, together with the most frequent inspection findings, can be useful in setting training priorities for the shipping industry.

It seems that water safety monitoring was the most important problem found, followed by inappropriately equipped or absent hand washing facilities in galleys, and inadequate or absent sharps or biomedical collectors. In Belgium, 48% of the samples tested were legionella–positive (Dirk Van Reusel, personal communication). These findings can cause serious public health risks. Legionnaires’ disease cases have been reported in both cargo and passenger ships, including fatal cases [15,16,17]. International conventions and European legislation require seafarers to be trained or certified as competent or qualified to perform their duties. Both the Maritime Labour Convention (MLC) and the European Directive 2009/13/EC, implementing the Agreement concluded by the European Community Shipowners’ Associations (ECSA), and the European Transport Workers’ Federation (ETF) on the Maritime Labour Convention (MLC 2006) include specific provisions for training of seafarers on health and hygiene issues and for ongoing training [18,19]. Both legal documents require that on-board programmes for the prevention of diseases among seafarers must be in place, while certain training standards are required for cooks and catering staff of ships according to the Flag State. Training providers can consider guidelines given in various WHO documents when designing training courses for the shipping industry [5,20,21,22].

Belonging to ship categories “C” and “E” (Container ships and Other Activities and Fish Catching and Other Fishing), as well as having a flag of one of the EU MS, were protective factors in having ≥1 inspection findings. Contrarily, belonging to ship categories “A” (Bulk Dry, Bulk Dry/Oil, Other Bulk Dry, Other Dry Cargo, Self-Discharging Bulk Dry) and “G” (Inland Waterways Dry Cargo/Passenger and Inland Waterways Tanker), as well as having empty cargo holds at the time of inspection were contributing factors in having an SSCC. Belonging to ship category “J” (Chemical) was a contributing factor in having ≥1 inspection findings. Ideally, cargo holds can be better inspected when they are empty, in order for inspectors to access and inspect all areas. However, depending on the ship type, inspection of cargo holds can be challenging and requires special safety precautions. Moreover, logistic regression analysis showed that a factor contributing to the holding of an SSCC was ship age (≥12 years). Older ships are expected to have construction deficiencies due to damage and failure in equipment. These could be a reason that older ships most frequently received an SSCC. These results can be useful to competent authorities for prioritizing ship inspections and possibly including announced inspections on ships presenting a high threat for public health risks. In addition, the results can be useful for shipping companies to improve their performance.

Data collection from NFPs through the situation analysis survey was conducted prior to the EIS development in 2010. Since then, policies and practices may have changed taking into consideration the efforts of EU MS in implementing IHR (2005) and Decision No. 1082/2013/EU of the European Parliament and the Council of 22 October 2013 on serious cross-border threats to health, as well as the WHO Handbook for ship inspections [3,5,23]. However, all Member States showed an interest in using a European database to record the results of ship inspections and for the issuance of SSCECs/SSCCs. According to Member States, the main benefits from such a database would be: (1) the ability to consult previous inspection reports and previous SSCECs/SSCCs; and (2) to produce inspection reports and SSCECs/SSCCs [7]. The EIS developed has been systematically used by specific ports in 11 countries. It is expected that additional ports will use the EIS in the future, considering that ten EU MS have enacted Ministerial Circulars or other internal rules, incorporating the EIS into their national practices [24]. Further efforts in the form of dissemination activities, including publications and messages in existing professional networks (such as national professional associations) and international networks (such as the WHO Ports, Airports, and Ground Crossing Network), could increase the use of the EIS [25].

## 5. Conclusions

Our study analysed data recorded in an inter–country information system for issuing SSCs. In addition, the study examined the future potential for additional countries and inspectors who will use the EIS. The EIS would allow inspectors the opportunity to monitor trends over time, such as improvements in ships’ performance. These opportunities could be examined once a higher number of inspections are recorded in the EIS in the future. Analysis of results per inspector could be useful for improving work performance and setting training priorities, considering the available WHO learning programmes [6].

Although one function of the SSC is to serve as a communication tool among competent authorities at ports, the possibility of having an electronic platform available would facilitate information sharing in such a way that inspectors will be prepared before the ship arrives to their port. The EIS could serve as a basis to develop similar systems in other countries. This will diminish the practices that have occurred in the past, where SSCs recording public health risks were destroyed and a new certificate was asked to be issued in the next port of call. Our study presents the analysis results of more than 10,000 SSCs under the IHR (2005), recorded in the EIS that was developed to record SSCs by EU MS-competent authorities. It reveals areas where shipping companies and competent authorities should focus on improving health and hygiene on ships sailing internationally. The study further proves the usefulness of recording SSC results in an inter-country information system; as more inspectors have access to the system, a greater number of inspections can be followed up, and the health and hygiene performance of ships can be monitored and improved. Further study needs have been raised in regards to knowledge, attitudes, and practices of stakeholders, in order to better understand how the IHR (2005) is implemented and to plan for improvements, especially through training. In the future, a global information system, or connection of national or regional information systems and exchanges of data could help to better implement the SSCs using common standards and procedures.

## Figures and Tables

**Table 1:** Descriptive characteristics of ships and summary inspection findings.

Characteristics		Frequency (%)
Ship flag	EU MS	5026 (47.0)
	non-EU MS	5658 (53.0)
	Total	10,684
Ship age	≥12	4002 (38.2)
	<12	6479 (61.8)
	Total	10,481 (100.0)
Ship type	A	1622 (15.3)
	B	69 (0.6)
	C	919 (8.6)
	D	19 (0.2)
	E	3878 (36.5)
	F	9 (0.1)
	G	791 (7.4)
	H	8 (0.1)
	I	1423 (13.4)
	J	145 (1.4)
	K	1747 (16.4)
	Total	10,630 (100.0)
Loaded with cargo at the time of inspection	No	2364 (22.1)
	Yes	8334 (77.9)
	Total	10,698 (100.0)
Inspection with at least one finding in inspection areas	Yes (>0 area)	3760 (35.6)
	No	6800 (64.4)
	Total	10,560 (100.0)
Number of inspections with 0 or >0 findings	Yes (>0 finding)	3482 (33.0)
	No (0 finding)	7078 (67.0)
	Total	10,560 (100.0)
Number of inspections with 0, 1, >1 findings	>1 finding	1578 (14.9)
	1 finding	1904 (18.0)
	0 finding	7078 (67.0)
	Total	10,560 (100.0)
Type of SSC issued	SSCC	296 (2.8)
	SSCEC	10,281 (97.2)
	Total	10,577 (100.0)

**Table 2:** The 10 most frequent inspection findings in all inspections and in inspections where an SSCC was issued.

The 10 Most Frequent Inspection Findings in All Inspections N = 7565		
Item Number According to the WHO Handbook [5]	Item Description	Frequency of the 10 Most Cited Findings (%)
9.1.1	No water quality analysis report available, last analysis report shows contamination or not all required parameters have been analysed ^1^	1706 (23)
2.2.1	Hand-washing station in the galley absent or inadequately equipped	381 (5)
5.2.3	Absence or inadequate sharps or biomedical collectors	370 (5)
3.2.1	Soiled stores	327 (4)
3.4.3	Foods found in contact with the deck, standing water or other contamination	280 (4)
2.7.2	Evidence of improper cleaning procedures and improper use of cleaning chemicals and disinfectants	199 (3)
2.1.3	No routine cleaning programme and schedule	194 (3)
5.2.2	Paper towels or hand-drying device, liquid soap, waste receptacle, toilet brush or toilet paper missing	191 (3)
2.8.1	Food handlers do not demonstrate competencies concerning hygiene	163 (2)
2.4.4	Perishable foods found stored at incorrect temperatures for the type or class of food. If time control used, no explanation or documentation for periods longer than 6 h	161 (2)
**The 10 Most Frequent Inspection Findings in Inspections Where a SSCC Was Issued N = 1496**		
9.1.1	No water quality analysis report available, last analysis report shows contamination or not all required parameters have been analysed ^1^	116 (7.8)
2.1.3	No routine cleaning programme and schedule	71 (4.7)
3.2.1	Soiled stores	66 (4.4)
3.4.3	Foods found in contact with the deck, standing water or other contamination	54 (3.6)
2.2.1	Hand-washing station in the galley absent or inadequately equipped	52 (3.5)
2.7.7	Evidence of vector infestation	44 (2.9)
2.1.4	No temperature logs for received goods, freezers, cold storage, holding temperatures or preparation temperatures. No calibrated thermometers available	43 (2.9)
2.7.4	Evidence of accumulated soil and grease on previously cleaned food contact surfaces	35 (2.3)
5.2.3	Absence or inadequate sharps or biomedical collectors	35 (2.3)
2.2.3	Food contact surfaces, utensils and equipment not durable, corrosion resistant and non-absorbent	32 (2.1)

^1^ In the majority of those inspections, Captains requested inspectors to collect water samples, in order to obtain water quality reports.

**Table 3:** Frequency of inspection findings per ship area.

Inspection Area as Per WHO Handbook [5]	Inspection Findings		Inspections with at Least One Finding in the Inspection Area				
	Number (%)	Mean (Standard Deviation)	SSCEC	SSCC	Extension	Inspection without SSC	Total (%)
Area 1: Quarters	163 (2%)	0.0 (0.2)	73	53	2	0	128 (3%)
Area 2: Galley, pantry and service areas	2569 (34%)	0.3 (0.9)	1287	206	0	2	1495 (40%)
Area 3: Stores	1168 (15%)	0.1 (0.5)	782	147	1	2	932 (25%)
Area 4: Child-care facilities	1 ^1^ (0%)	-	2	0	0	0	2 (0%)
Area 5: Medical facilities	868 (11%)	0.1 (0.4)	583	97	1	2	683 (18%)
Area 6: Swimming pools and spas	4 ^1^ (0%)	0.0 (0.1)	5	1	0	0	6 (0%)
Area 7: Solid and medical waste	102 (1%)	0.0 (0.1)	56	32	1	0	89 (2%)
Area 8: Engine room	0 ^1^ (0%)	-	1	0	0	0	1 (0%)
Area 9: Potable water	2586 (34%)	0.3 (0.6)	2079	189	2	15	2285 (61%)
Area 10: Sewage	24 (0%)	0.0 (0.1)	17	7	0	0	24 (1%)
Area 11: Ballast water	0 ^1^ (0%)	-	0	1	0	0	1 (0%)
Area 12: Cargo holds	4 ^1^ (0%)	0.0 (0.0)	4	1	0	0	5 (0%)
Area 13: Other systems and areas	76 ^1^ (1%)	0.0 (0.1)	46	31	1	0	78 (2%)
Total findings/inspections with evidence found	7565	-	3449	292	3	16	3760

^1^ Inspections recorded with evidence found, but no item recorded in the European Information System (EIS).

**Table 4:** Univariate analysis of factors in relation to Ship Sanitation Control Certificates (SSCCs) and to the number of inspection findings.

Factors	Relative Risk (RR), Confidence Intervals (CI) and Predictive Values (*p*-Values)					
	Inspection with an SSCC			Inspections with ≥1 Findings		
	RR	95% CI	*p*-Value	RR	95% CI	*p*-Value
Ship flag (EU MS versus non-EU MS)	1.04	0.83–1.30	0.753	0.76	0.71–0.80	<0.001
Ship age (≥12 versus <12)	1.75	1.39–2.19	<0.001	0.98	0.92–1.03	0.432
>1 area with at least one inspection finding	175.34	56.26–546.42	<0.001	N/A	N/A	N/A
Absence of cargo at cargo holds at the time of inspection versus presence of cargo	2.53	2.02–3.18	<0.001	1.12	1.09–1.16	<0.001
Number of inspection findings (≥1 versus no findings)	14.12	10.03–19.88	<0.001	N/A	N/A	N/A

N/A: not applicable.

**Table 5:** Logistic regression analysis of factors in relation to Ship Sanitation Control Certificates (SSCCs) and to the number of inspection findings.

Factors		Odds Ratio (OR), Confidence Intervals (CI) and Predictive Values (*p*-Values)					
		Inspection with an SSCC			Inspections with ≥1 Findings		
		OR	95% CI	*p*-Value	OR	95% CI	*p*-Value
Ship flag (EU MS versus non-EU MS)	EU	N/A	N/A	N/A	0.72	0.66–0.79	<0.001
Ship age (≥12 versus <12)	≥12	1.77	1.37–2.29	<0.001	N/A	N/A	N/A
Ship type ^1^ (versus category K)	A	2.30	1.35–3.92	0.002	0.87	0.76–1.01	0.059
	B	1.91	0.46–7.99	0.373	0.63	0.35–1.11	0.111
	C	1.67	0.85–3.27	0.139	0.50	0.41–0.61	<0.001
	E	1.64	0.99–2.72	0.056	0.85	0.75–0.96	0.008
	G	2.50	1.38–4.52	0.002	1.23	1.03–1.46	0.022
	I	1.58	0.87–2.87	0.130	1.01	0.87–1.17	0.921
	J	0.83	0.23–3.00	0.777	1.51	1.07–2.13	0.018
Absence of cargo at cargo holds at the time of inspection versus presence of cargo	Absence of cargo	3.36	2.51–4.50	<0.001	N/A	N/A	N/A
Number of inspection findings (>1 and 1 versus no findings)	> 1 finding	35.83	24.76–51.84	<0.001	N/A	N/A	N/A
	1 finding	3.51	2.12–5.81	<0.001	N/A	N/A	N/A

^1^ Categories D, F, & H were excluded due to the low numbers of inspections on those ship categories N/A: not applicable.
